# *C4BPA*: A Novel Co-Regulator of Immunity and Fat Metabolism in the Bovine Mammary Epithelial Cells

**DOI:** 10.3389/fgene.2021.830566

**Published:** 2022-01-31

**Authors:** Ambreen Iqbal, Pan Ziyi, Haibin Yu, Li Jialing, Wu Haochen, Fan Jing, Jiang Ping, Zhao Zhihui

**Affiliations:** ^1^ Department of Animal Sciences, College of Coastal Agricultural Sciences, Guangdong Ocean University, Zhanjiang, China; ^2^ College of Animal Science and Technology, Anhui Agricultural University, Hefei, China

**Keywords:** C4BPA, bMECs, TLR-4/NF-κB, complement, fat metabolism

## Abstract

The C4b binding protein alpha (C4BPA) chain primarily engages in critical inflammatory and coagulation processes. The previous transcriptomic analysis showed that *C4BPA* is a differentially expressed gene in lower and higher fat content mammary gland cell lines from Chinese Holstein. This study aimed to investigate the effects of *C4BPA* on the inflammation and milk fat synthesis in bMECs by *C4BPA* knockdown and overexpression. The results highlighted that knockdown of *C4BPA* in bMECs could suppress the mRNA and protein expression of *IL-6, IL-8, IL-12*, and the TLR-4/NF-κB pathway-related genes and promote the expression of complement and coagulation cascade pathways related genes as well as *TNF-α*. Moreover, knockdown of *C4BPA* expression in bMECs reduced the content of triglyceride (TG) and cholesterol (CHOL) in bMECs, increased NEFA content, reduced mRNA and protein expression of *ACSL1* and *PPARA*, and increased the mRNA and protein expression of *ELOVL6*, *FADS1*, and *LPL*. The bMECs, with the overexpression of *C4BPA*, showed the enhanced expression of *TLR-4/NF-κB* linked genes, *IL-6*, *IL-8*, *IL-12*, and mRNA and protein level while reduced mRNA expression of *TNF-α*, compliment, and coagulation cascade related genes was observed. In bMECs, overexpression of *C4BPA* enhanced the content of TG and CHOL while reducing NEFA and stimulated the mRNA and protein expression of *ACSL1*, *PPARA*, and *PPARG* genes while inhibiting the mRNA and protein expression of *FADS1* and *LPL* genes. Our results show that *C4BPA* not only regulates the lipid metabolism through the PPAR signaling pathway in bMECs but also contributes to the inflammatory response through TLR-4/NF-κB and the complement and coagulation cascade pathways. This study, for the first time, provides the primary basis for understanding the role of *C4BPA* in immunity and fat metabolism, which enables the researchers for innovative direction to investigate genes associated with fat metabolism and immunity. This study also advocates that the breeders must pay attention to such type of genes with multiple functions during animal breeding.

## Introduction

Milk is a high fat, protein, vitamin, and other nutrient-rich liquid food secreted from the mammalian glands as a key nourishment source for infants of mammals (Winckel et al., 2011). The primary milk fat components, followed by fatty acids and cholesterol, are triglycerides (Leonard et al., 2004). Because of the enrichment of nutrients in milk, humans also consume it to meet their dietary needs. Cow milk is the largest contributor to the world’s milk output in commercial production (Gerosa and Skoet 2012).

*C4BPA* is one of the genes we found to function in lipid metabolism traits, and this gene is related to inflammation. Several diseases lead to inflammation of the udder, including mastitis. Mastitis is one of the most common and destructive diseases for dairy cattle. Mastitis is an inflammatory reaction in the bovine mammary tissue induced by infection with pathogens or other physical and chemical causes. Dairy cow mastitis is the consequence of issues related to genetics, environment, and food management. It can lead to a decline in milk quality and quantity, a decrease in growth and development of the animal, and a decrease in fertility, leading to significant economic losses and entailing the development of the dairy industry (Waseem Ghani et al., 2019). In order to cope with this issue, breeding of dairy cows that are resistant to mastitis is essential. Several studies have been conducted over the past few years to collect data on the genetic factors and breeding values of dairy cows with mastitis (Martin et al., 2018). Many anti-mastitis potential genes are investigated by genome-wide association studies (GWAS). Using *E. coli* infected cattle’s mammary tissues, Mitterhuemer identified 2,754 and 476 differential genes in the mammary tissues of the mastitis cattle. These genes included antifungal genes (*S100A8*, *S100A9*, *S100A12*, *CXCL2*, *GNLY*), acute-phase genes (*LBP*, *SAA3*, *CP*, *BF*, *C6*, *C4BPA*, *IF*), and oxidative stress genes (*LBP*, *SAA3*, *GPX3*, *MT1A*, *MT2A*, *SOD2*) (Mitterhuemer et al., 2010).

The *C4BPA* gene is located on chromosome 1q32 and contains 12 exons and 11 introns. The soluble alpha chain of *C4BPA* is a major inhibitor of the conventional complement pathway (Suankratay et al., 1999). Three types of *C4BP* are found: α7β1, α7β0, and α6β1 (Scharfstein, Ferreira, and Gigli 1978). In all three forms, α7β1 is the more abundant while α6β1is inconsequential (Moffat et al., 1992). C4BP has various isoforms, and the one called C4BPA is generally expressed at higher levels during inflammation. In the C4BP alpha chain, the binding sites are found for heparin (Hessing M, 1990), low-density lipoprotein receptor-related protein LRP (Westein et al., 2002), C3b, serum amyloid protein (Garcia de Frutos P and Dahlbäck B 1994), and the surface proteins of various bacteria (Blom AM, 2000; Ram S, 2001), which are the master player in lipid metabolism, inflammation, and coagulation pathways (Ridker et al., 2008; Antoniades et al., 2009).

A previous study also reported that the C4BPA possesses the binding site for heparin, C-reactive protein, and CD40, the specific key mediators during the inflammation process and the blood coagulation pathways (Antoniades et al., 2009). In human B cells, the C4BPA directly binds with CD40 at the various positions of the CD40 ligand. The vital role of C4BPA is in the induction of the proliferation of B cells and the activation of CD40, which in turn stimulates the anti-tumor response of T cells (Brodeur et al., 2003). Sogawa et al. reported that the levels of C4BPA was found to be higher in the serum of pancreatic ductal adenocarcinoma (PDAC) patients compared to the normal human serum (Sogawa et al., 2016). Due to the significant binding ability of C4BPA with CD40, the C4BPA is a key factor involved in dairy cattle mastitis. The research on mastitis development reported that the inflammatory factors including *IL-6*, *IL-8*, *TNF-ɑ*, and *TLR4* are consistently altered (Harada et al., 1994; Strandberg et al., 2005; Fu et al., 2013).

The previous transcriptomic analysis of low- and high-fat milk-producing Chinese Holstein cattle showed that *C4BPA* is a differentially expressed gene with higher expression in high-fat milk cattle (Xia et al., 2021). However, the co-regulatory role of the *C4BPA* gene against inflammation and fat metabolism in Chinese Holstein cattle is still unknown. Therefore, this study investigates the key role of the *C4BPA* gene in milk fat synthesis and inflammation through its overexpression and knockdown in bMECs. In order to delineate the aforementioned co-regulatory role of *C4BPA*, the different regulators and indicators of inflammation and milk fat were analyzed in different experimental groups. This study provides the major basis for breeding cattle with high resistance to mastitis and good milk quality through the proper knowledge of the molecular mechanism of such type genes. According to our best knowledge, this is the first study exploring the co-regulatory role of the *C4BPA* in milk fat metabolism and mastitis.

## Materials and Methods

### Cell Source

The animal genetics and breeding laboratory at Jilin University kept BMECs from Chinese Holstein dairy cows (Lu et al., 2012). All studies were conducted under Jilin University’s rules for the care and use of experimental animals (permission number: SY201901007 (Jiang et al., 2020).

### Construction of *C4BPA* Interference and Overexpression Plasmids

NCBI identification of a CDS-specific fragment, four C4BPA-shRNA interference sequences (pGPU6-C4BPA-shRNA1, pGPU6-C4BPA-shRNA2 pGPU6-*C4BPA*-shRNA3, and pGPU6-*C4BPA*-shRNA4), and negative control (shNC) were designed and synthesized. The sequence is shown in Table 1. Interfering RNA (shRNA) oligonucleotides were annealed and cloned into the pGPU6-GFP-Neo knockdown vector (C02007, Genepharma Corporation, Shanghai, China). Annealing system (10 µl): 1 µl each of the upper and lower primers, 10X annealing buffer 1, and 7 µl ddH2O were incubated in the PCR apparatus at 95°C for 5 min, then slowly annealed to room temperature, and stored at 4°C for later use.

**TABLE 1:** List of four shRNA sequences.

Name	Sequence (5'—3′)	
pGPU6-*C4BPA*-shRNA1	F	CAC​CGG​TTT​GTA​TTG​AGC​TAC​AAG​TTT​CAA​GAG​AAC​TTG​TAG​CTC​AAT​ACA​AAC​CTT​TTT​TG
	R	GAT​CCA​AAA​AAG​GTT​TGT​ATT​GAG​CTA​CAA​GTT​CTC​TTG​AAA​CTT​GTA​GCT​CAA​TAC​AAA​CC
pGPU6-*C4BPA*-shRNA2	F	CAC​CGG​ACC​TAC​AAC​TGT​GAC​TTG​TTT​CAA​GAG​AAC​AAG​TCA​CAG​TTG​TAG​GTC​CTT​TTT​TG
	R	GAT​CCA​AAA​AAG​GAC​CTA​CAA​CTG​TGA​CTT​GTT​CTC​TTG​AAA​CAA​GTC​ACA​GTT​GTA​GGT​CC
pGPU6-*C4BPA*-shRNA3	F	CAC​CGG​CAC​TTG​GAG​TCC​TAG​AAC​ATT​CAA​GAG​ATG​TTC​TAG​GAC​TCC​AAG​TGC​CTT​TTT​TG
	R	GAT​CCA​AAA​AAG​GCA​CTT​GGA​GTC​CTA​GAA​CAT​CTC​TTG​AAT​GTT​CTA​GGA​CTC​CAA​GTG​CC
pGPU6-*C4BPA*-shRNA4	F	CAC​CGC​TTA​CCA​CAC​ATC​CCT​CAT​GTT​CAA​GAG​ACA​TGA​GGG​ATG​TGT​GGT​AAG​CTT​TTT​TG
	R	GAT​CCA​AAA​AAG​CTT​ACC​ACA​CAT​CCC​TCA​TGT​CTC​TTG​AAC​ATG​AGG​GAT​GTG​TGG​TAA​GC

The coding region of *C4BPA* in the cow is amplified with forwarding primer 5′- GGA​TCCATG​AAG​CAT​CAG​CGA​GTT​C-3′ (the underlined sequence indicates a BamHI) and reverse primer 5′- ATC​GATCTA​TTC​TGG​ATT​AAA​GTC​ACA​AGT​CA -3′ (the underlined sequence indicates a ClaI). Then, PCR was used to clone an 1833 bp product, followed by its purification. The *C4BPA* sequence, containing BamHI (#R3136L, NEB, China) and ClaI (#R0197V, NEB, China) restriction endonuclease cleavage sites, was recombined and ligated into the pBI-CMV3 expression vector (#631632, Clontech Laboratories, Mountain View, CA). Finally, it was confirmed by sequencing to obtain the *C4BPA* overexpression vector (pBI-CMV3-*C4BPA*). The Shanghai Shenggong Biotechnology Co., Ltd. constructed the plasmid, and the point mutation kits were used for site-directed mutagenesis (#C215-01, Vazyme, China).

### Cell Culture and Transfection

The bMECs were cultured in culture plates (353003, Falcon, Franklin Lakes, NJ) in Dulbecco’s modified Eagle medium with nutrient mixture F12 (12-719Q, HyClone, Logan, UT) supplemented with 10% fetal bovine serum (11,011-6,123, Tian Hang, Zhejiang, China), with 1% antibiotics (SV30010, HyClone). Cells were transferred into six-well culture plates (353090, Falcon) at a concentration of 2 × 10^5^ cells/well and cultured at 37°C and 5% CO_2_ in an incubator (Thermo Fisher Scientific, Waltham, MA). When cells reached 80% confluence, the cell culture medium was changed to a medium without antibiotics for transfection (Jiang et al., 2018). According to the manufacturer’s protocol, the various vectors were transfected into cells using a FuGENE HD Transfection Reagent (PRE2311, Promega, Madison, WI). Briefly, the mixture of 3 µg of plasmid DNA and 7.5 µl of FuGENE HD in 150 µl of Opti-MEM (Thermo Fisher Scientific) was incubated at room temperature for 15 min added into the culture medium in each well of bMECs. After 24 h, the expression of green fluorescent protein (GFP) in cells was observed under a fluorescence microscope (TE 2000, Nikon, Tokyo, Japan) to determine the transfection efficiency. Three replicates of the experiments were carried out by transfecting equal numbers of cells using the same vectors in the various wells.

### Real-Time Quantitative Reverse Transcription Polymerase Chain Reaction

Cells transfected with pGPU6-*C4BPA*-shRNA1 and the pBI-CMV3-*C4BPA* were harvested 24 h after transfection. Then, 1 μg of total RNA was extracted with an RNA extraction kit (Analytik Jena, Jena, Germany). The concentration and purity of the RNA were determined using a spectrophotometer, further confirmed *via* 1% agarose gel electrophoresis. The cDNA was obtained using a cDNA synthesis kit (RR047A, TaKaRa Biotechnology, Dalian, China). Then, real-time quantitative reverse transcription polymerase chain reaction (qRT-PCR) was performed with SYBR Premix Ex Taq (RR420L, TaKaRa Biotechnology). The primers’ sequences are shown in Table 2. We performed PCR amplification in 20 µl reaction volume under the following conditions: an initial denaturing step of 95°C for 30 s, followed by 40 cycles of 95°C for 10 s, and then 60°C for 45 s. All samples were adjusted to the same concentration. The mRNA expression was measured *via* qRT-PCR with *β*-actin as the reference gene for internal control. All the experiments of qRT-PCR were repeated at least three times.

**TABLE 2:** List of primer sequences used for qRT-PCR.

Genes	-	Primer sequences (5′-3′)	Product (bp)	Annealing temperature (°C)
*C4BPA*	F	TGA​AGC​ATC​AGC​GAG​TTC​CAG​T	249	58.5
	R	AGC​CAG​GAC​GAC​AGG​TGT​ATC​T		
*TLR4*	F	GCA​GGG​AAA​GTC​AAC​TAA​AC	225	60
	R	ACA​TAA​AGT​GGA​GGG​GAA​TC		
*IL-6*	F	AGG​CAG​ACT​ACT​TCT​GAC​CA	232	52
	R	TAC​TCC​AGA​AGA​CCA​GCA​GT		
*IL-8*	F	CTG​CAG​TTC​TGT​CAA​GGA​TG	201	57
	R	CAA​CCT​TCT​GCA​CCC​ACT​TT		
*TNF-α*	F	CAG​TCT​CCT​ACC​AGA​CCA​AG	186	53
	R	CAG​CAT​AGT​CCA​GGT​AGT​CC		
*IL-10*	F	CCT​TGT​CGG​AAA​TGA​TCC​AG	115	56
	R	CGC​AGG​GTC​TTC​AGC​TTC​T		
*C1S*	F	GGA​CGG​AGG​ACC​AAC​AAG​AA	226	60
	R	GGC​AGG​AGC​AGA​AGT​AAC​CA		
*C2*	F	CTG​AAC​CTC​TAC​CTG​CTC​CTG	130	55
	R	TGA​TGG​CGA​CAC​TAA​CCT​TGA		
*C3*	F	CAG​AAG​GCG​TAA​GAG​TCA​ACA​A	188	60
	R	GAT​GGC​GTC​CTC​CGT​CAT​T		
*C3A*	F	ATG​GTG​GTG​GTG​GTT​GTC​TT	177	60
	R	CAG​GAG​AGC​ATA​GAG​GAA​TGG​A		
*C4A1*	F	GAAGGGGGAGGATGAGCA	221	60
	R	CGC​TCC​CAT​CTG​TGT​TCT​G		
*ACSL-1*	F	ATA​AAG​ACG​GCT​GGT​TGC​AC	154	60
	R	GGT​TCA​CTC​CGC​TGG​TAG​A		
*FADS1*	F	GCT​CGG​GAA​ACA​GAA​GAA​A	154	60
	R	AGG​AAG​ATA​CGG​ACG​TAG​AAA​G		
*LPL*	F	GAACTGGATGGCGGATGA	182	60
	R	GAG​AAA​GGC​GAC​TTG​GAG​C		
*ACADS*	F	GTC​GCC​TAC​ATA​CCG​TCT​ACC	160	60
	R	TCGCCCATCTTCTTCACC		
*PPARγ*	F	AGC​ATT​TCC​ACT​CCG​CAC​TA	125	60
	R	GGG​GAT​ACA​GGC​TCC​ACT​TTG​AT		
*PPARɑ*	F	CAACCCGCCTTTCGTCAT	106	60
	R	GACTTCCGCCTCCTTGT		
*ELOVL6*	F	AGC​CTT​TAG​TGC​TCT​GGT​CTC	193	60
	R	CCT​AGT​TCG​GGT​GCT​TTG​C		
*β-Actin*	F	CAT​CGG​CAA​TGA​GCG​GTT​C	144	60
	R	GTG​TTG​GCG​TAG​AGG​TCC​TT		

### Triglyceride, Cholesterol, and Non-Eesterified Free Fatty Acid Detection

Cells transfected with pGPU6-*C4BPA*-shRNA1 and pBI-CMV3-*C4BPA* were harvested 24 h after transfection. A blank control group was set as the reference group using double-distilled water. Triglyceride and total cholesterol content were measured using a TG and CHOL detection kit (E1015-105 and E1013, Applygen Technologies, Beijing, China). Non-esterified free fatty acid content was measured using a NEFA detection kit (A042-2-1, Nanjing Jiancheng Bioengineering Institute, China). Each microgram of protein adjusted the cellular contents of TG, CHOL, and NEFA by the BCA (E112-01, Vazyme, China) protein quantitative analysis method. The concentrations were measured using a microplate reader (SM600, Shanghai YongChuang Medical Instrument Co. Ltd., Shanghai, China)

### Western Blot

Cells transfected with pGPU6-*C4BPA*-shRNA1 and pBI-CMV3-*C4BPA* were harvested 24 h after transfection. After 24 h of transfection, trypsinize the cells and wash them three times with PBS. The cells were then resuspended in RIPA buffer with protease inhibitors (MA0151, Meilunbio). The cell lysate was removed by centrifugation at 12,000 rpm/min for 10 min at 4°C, and the supernatant was collected. The BCA protein quantitative analysis method determines the sample’s total protein concentration. For Western blot (PowerPac™ HV, Bio-Rad), separate the same amount of protein by SDS-PAGE and then transfer it to PVDF (88518, Thermo-Fisher) membrane with a membrane transfer machine. After blocking with 1×TBS buffer containing 1% BSA for 2 h, wash the PVDF membrane with TBST (B040126, Sangon Biotech, China) solution. Then, the membrane was incubated with a primary antibody at 4°C for 12 h. Incubate the membrane with a secondary antibody (RS0002, Immunoway) (diluted in 1% BSA) for 2 h. The enhanced chemiluminescence HRP substrate was used to observe protein bands. After taking a photo with a chemiluminescence instrument, grayscale analysis software (Tanon) was used to calculate the relative expression of the target protein in different samples. Primary antibodies were purchased from Bioss, China.

### Statistics and Analysis

Experimental data are shown as mean ± standard error (MSE) of the mean. Statistically significant differences are shown as *p < 0.05*. GraphPad Prism 9 software (GraphPad Software Inc., La Jolla, CA) with a 2-tailed (unpaired) *t*-test used for the data analysis.

## Results

**FIGURE 1:**
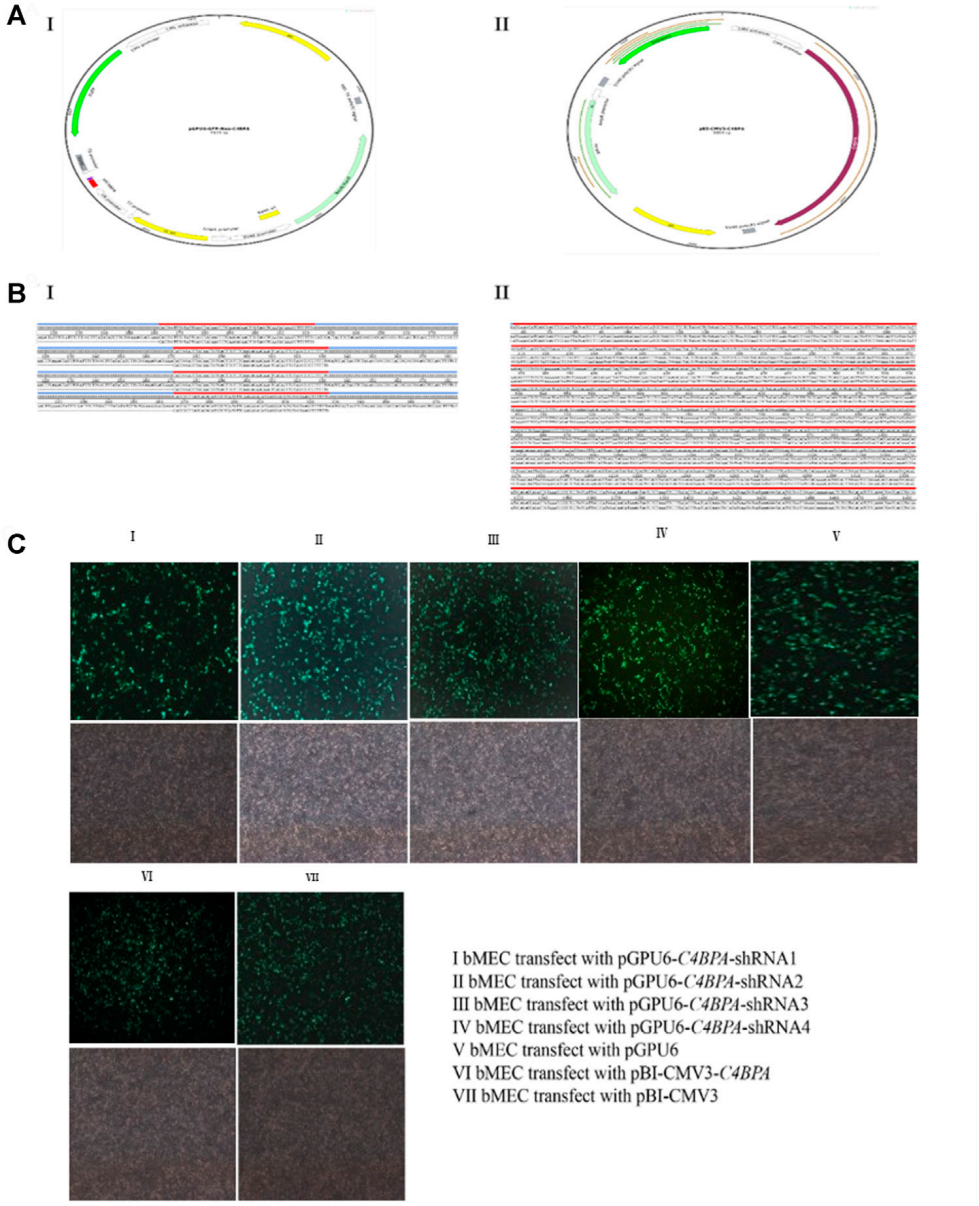
Construction of *C4BPA* gene interferences and overexpression vector. **(AI)** Construct an interference vector map. **(AII)** Construct C4BPA overexpression vector map. **(BI)** pGPU6-*C4BPA*-shRNA1, pGPU6-*C4BPA*-shRNA2, pGPU6-*C4BPA*-shRNA3, and pGPU6-*C4BPA*-shRNA4 four interferences vector sequences. **(BI)** pBI-CMV3-*C4BPA* sequencing. **(CI)** bMECs transfected with pGPU6-*C4BPA*-shRNA1. **(CII)** bMECs transfected with pGPU6-*C4BPA*-shRNA2. **(CIII)** bMECs transfected with pGPU6-*C4BPA*-shRNA3. **(CIV)** bMECs transfected with pGPU6-*C4BPA*-shRNA4. **(CV)** bMECs transfected with pGPU6. **(CVI)** bMECs transfected with pBI-CMV3-*C4BPA*. **(CVII)** bMECs transfected with pBI-CMV3.

Sequencing and the transfection efficiency of pBI-CMV3-*C4BPA* and pGPU6-*C4BPA*-shRNAs in bMECs: the standard sequencing results of the shRNAs and overexpression vector of *C4BPA* are displayed in Figure 1 (Figures BI and BII). After the successful construction and harvesting, the pBI-CMV3-*C4BPA*, pGPU6-*C4BPA*-shRNA1, pGPU6-*C4BPA*-shRNA2, pGPU6-*C4BPA*-shRNA3, and pGPU6-*C4BPA*-shRNA4 vectors were transferred to the cell plate when the bMECs attained 80 percent cell confluency. After 24 h of transfection, the cell morphology and the expression of green fluorescence protein were observed under an inverted fluorescent microscope (Figure 1C). The results showed that transfection of knockdown and overexpression vectors was successfully achieved, and cell morphology of the bMECs remained unchanged.

### The Relative mRNA and Protein Expression of pGPU6-*C4BPA*-shRNAs in bMECs

**FIGURE 2:**
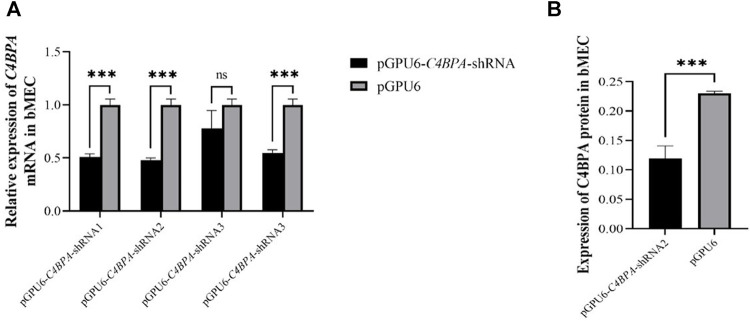
The relative expression of *C4BPA* at mRNA and protein level in different experimental groups of bMECs. **(A)** Relative mRNA expression of *C4BPA* gene after 24 h of transfection with pGPU6 *C4BPA*-shRNA1, pGPU6-*C4BPA*-shRNA2, pGPU6-*C4BPA*-shRNA3, pGPU6-*C4BPA* shRNA4, and pGPU6 in bMECs. **(B)** Relative protein expression of C4BPA after 24 h of transfection with pGPU6-C4BPA-shRNA2.

The bMECs were cultured in six well-plates. When the cells attained 80% growth, the *C4BPA* knockdown vectors, including pGPU6-*C4BPA*-shRNA1, pGPU6-*C4BPA*-shRNA2, pGPU6-*C4BPA*-shRNA3, pGPU6-*C4BPA*-shRNA4, and pGPU6, were transfected into the cells. After 24 h of successful transfection, the total RNA was extracted and reverse-transcribed and qRT-PCR was performed. The results showed that the mRNA expression of the *C4BPA* gene was significantly downregulated (*p < 0.001*) in pGPU6-*C4BPA*-shRNA1, pGPU6-*C4BPA*-shRNA2, and pGPU6-*C4BPA*-shRNA4 as compared to the pGPU6 (control group) in bMECs, which is shown in Figure 2A. When the bMECs were transfected with the pGPU6-*C4BPA*-shRNA3, the mRNA expression of the *C4BPA* did not show any significant downregulation compared to the pGPU6 shown in Figure 2A. Moreover, the protein expression of pGPU6-C4BPA-shRNA2 through ELISA was determined. The results showed that the pGPU6-C4BPA-shRNA2 was significantly downregulated (*p < 0.001*) compared to the pGPU6 (Figure 2A). Both of the above-mentioned results validate that pGPU6-*C4BPA*-shRNA2 significantly downregulated the mRNA and protein expression of the *C4BPA* gene. For further experiment, we selected the pGPU6-*C4BPA*-shRNA2 as the shRNA for the knockdown *C4BPA* gene.

### The Relative mRNA and Protein Expression of pBI-CMV3-*C4BPA* in bMECs

**FIGURE 3:**
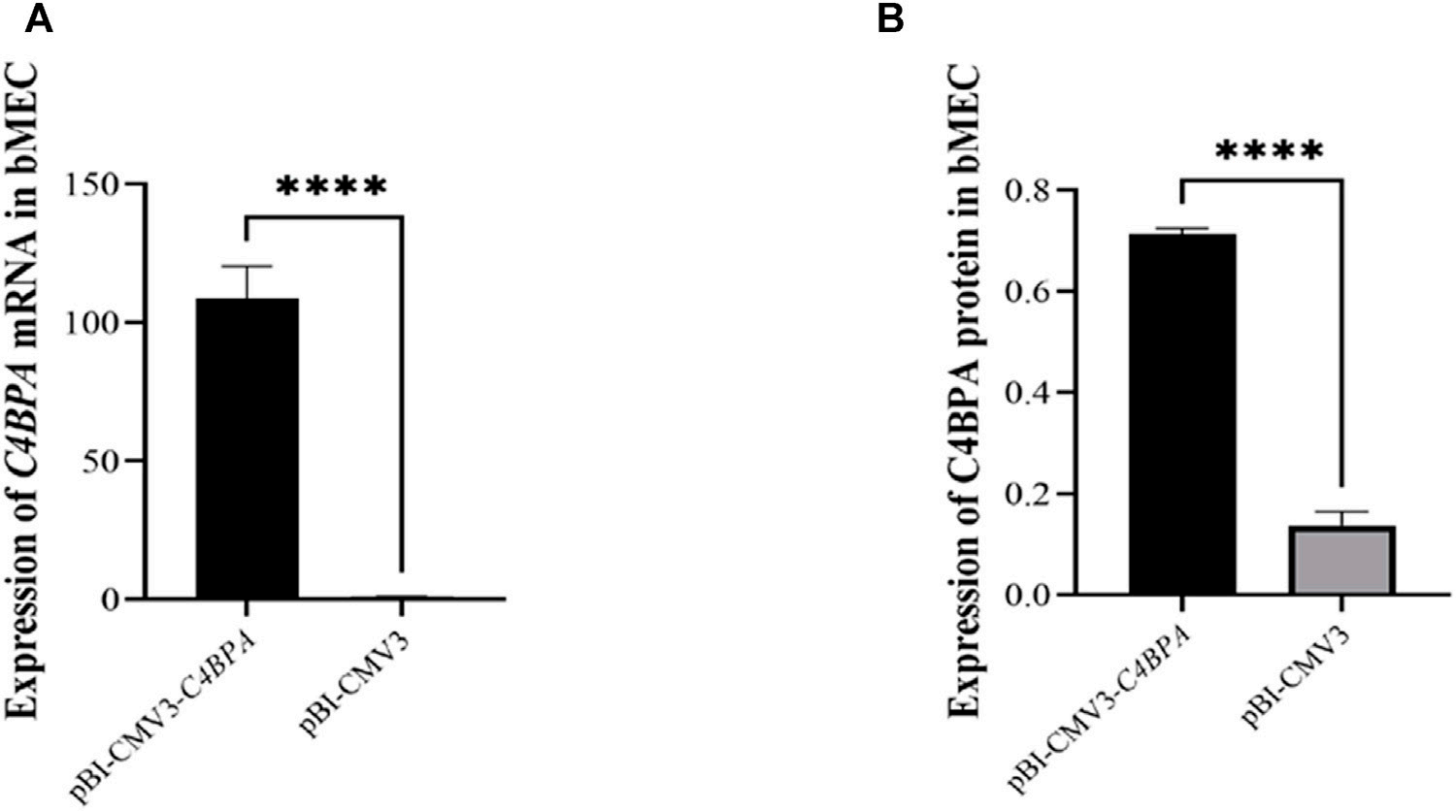
**(A)** Relative mRNA expression of *C4BPA* gene after 24 h transfection with pBI-CMV3-*C4BPA* and pBI-CMV3 in bMECs. **(B)** Relative protein expression of C4BPA gene after 24 h transfection with pBI-CMV3-C4BPA.

bMECs were cultured in six well-plates, and after reaching the 80% cell confluency, the shRNA vectors, including pBI-CMV3-*C4BPA* and pBI-CMV3, were transfected into the cells. After 24 h of successful transfection, qRT-PCR was performed. The result showed that the mRNA expression of the *C4BPA* gene was significantly upregulated (*p < 0.001*) in pBI-CMV3-*C4BPA* as compared to pBI-CMV3 in bMECs (Figure 3A). Moreover, the protein expression of C4BPA in bMECs transfected with pBI-CMV3-*C4BPA* determined through ELISA showed that pBI-CMV3-C4BPA was significantly upregulated (*p* < 0.001) compared to the bMECs transfected with pBI-CMV3 (Figure 3B). Taken together, these results validated that pBI-CMV3-*C4BPA* significantly upregulated the mRNA and protein expression of the *C4BPA* gene in bMECs.

### The mRNA Expression of *C4BPA* Influences the NF-κB Pathway-Related Genes in bMECs

**FIGURE 4:**
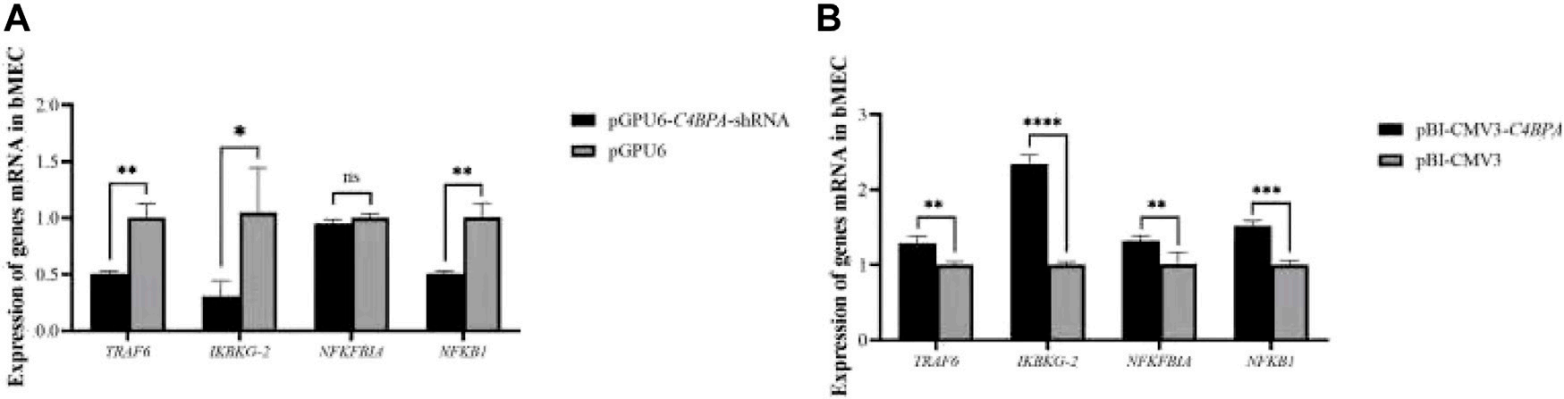
**(A)** The relative mRNA expression of NF-κB pathway-related genes in bMECs after transfection with pGPU6-*C4BPA*-shRNA2 vector. **(B)** The relative mRNA expression of NF-κB pathway-related genes in bMECs after transfection with pBI-CMV3-*C4BPA* vector.

The expression of the NF-κB pathway genes is investigated after the transfection of pGPU6-*C4BPA*-shRNA2 and pBI-CMV3-*C4BPA* vectors in bMECs. After 24 h of successful transfection, qRT-PCR was performed. The result showed that when bMECs are transfected with pGPU6-*C4BPA*-shRNA2, the genes that have a role in the NF-κB pathway, including the *TRAF6*, *IKBKG-2*, and *NF-κB1*, were significantly downregulated in the pGPU6-*C4BPA*-shRNA2 group as compared to pGPU6, which is shown in Figure 4A. The expression trend of *NFKFBIA* in the pGPU6-*C4BPA*-shRNA2 group had no significant downregulation trend compared to pGPU6, as shown in Figure 4A. The contrary result was obtained when bMECs were transfected with the pBI-CMV3-*C4BPA*. The genes involved in the NF-κB pathway, including *TRAF6*, *IKBKG-2*, *NFKFBIA*, and *NFKB1*, were all significantly upregulated in pBI-CMV3-*C4BPA* compared to pBI-CMV3, which is shown in Figure 4B. These results validated that *C4BPA* had a major influence on the NF-κB pathway genes and played a vital role in regulating these genes.

### The Relative mRNA Expression of Inflammatory Factors in bMECs With Knockdown and Overexpression of *C4BPA*

**FIGURE 5:**
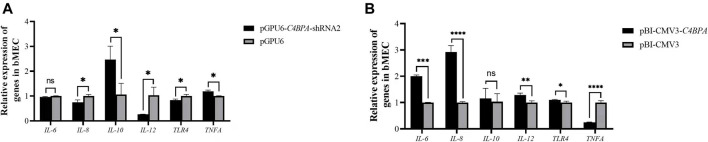
**(A)** The relative mRNA expression of inflammatory factors in bMECs after transfection with pGPU6-*C4BPA*-shRNA2 vector. **(B)** The relative mRNA expression of inflammatory factors in bMECs after transfection with pBI-CMV3-*C4BPA* vector.

The mRNA expression of the inflammatory factors was investigated by the transfection of the vectors pGPU6-*C4BPA*-shRNA2 and pBI-CMV3-*C4BPA* in bMECs. After 24 h of successful transfection, qRT-PCR was performed. The result showed that when bMECs are transfected with pGPU6-*C4BPA*-shRNA2, the genes with a role in the inflammatory factors, including *IL-8*, *IL-10*, and *IL-12*, were significantly downregulated (*p < 0.05*) in the pGPU6-*C4BPA*-shRNA2 group as compared to pGPU6 (Figure 4A). The expression trend of *IL-8* in the pGPU6-*C4BPA*-shRNA2 group had no significant downregulation trend compared to pGPU6, as shown in Figure 5A. The expression of *TNF-ɑ* in the pGPU6-*C4BPA*-shRNA2 group was significantly enhanced (*p < 0.05*) compared to pGPU6, as shown in Figure 4 A. The conflicting result was obtained when bMECs were transfected with pBI-CMV3-*C4BPA*. The genes involved in the NF-κB pathway, including inflammatory factors *IL-6*, *IL-8*, and *IL-12*, all were significantly upregulated in pBI-CMV3-*C4BPA* compared to pBI-CMV3, which is shown in Figure 5B. The expression trend of *IL-10* in the pBI-CMV3-*C4BPA* group had no significant downregulation trend compared to pBI-CMV3, as shown in Figure 5B. The expression of *TNF-ɑ* in the pBI-CMV3-C4BPA group was significantly downregulated (*p* < 0.05) compared to pBI-CMV3, as shown in Figure 5B. Altogether, these results validated that *C4BPA* had a major influence on inflammatory factors and played a vital role in regulating these genes.

### The Relative Protein Expression of C4BPA on Inflammatory Factors in bMECs

**FIGURE 6:**
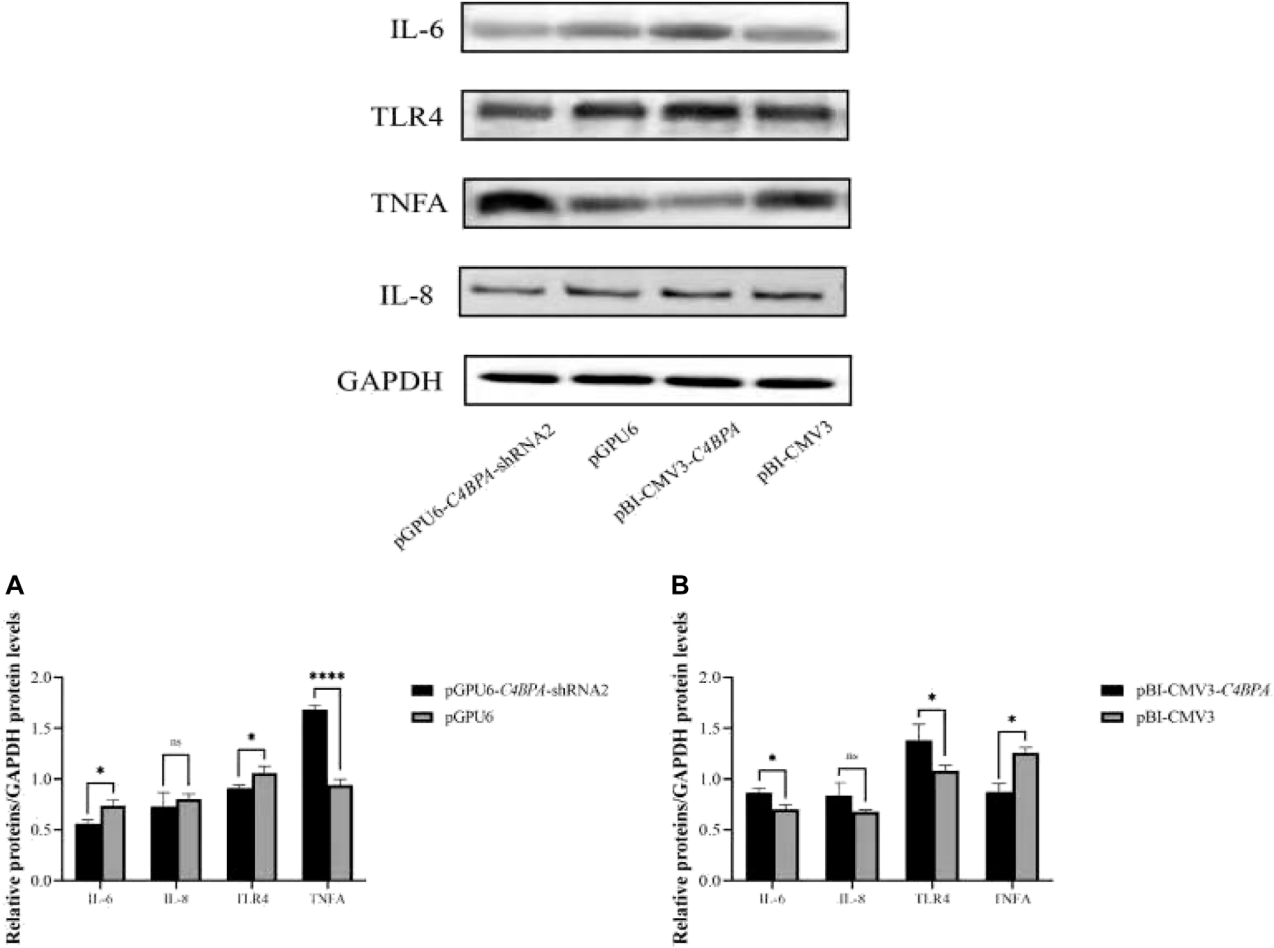
Relative protein expression of the immunity related factors in bMECs. **(A)** Relative protein expression of inflammatory factors in bMECs after transfection with pGPU6 C4BPA-shRNA2 vector. **(B)** Relative protein expression of inflammatory factors in bMECs after transfection with pBI-CMV3-C4BPA vector.

The protein expression of the inflammatory factors was explored by transfecting the vectors pGPU6-*C4BPA*-shRNA2 and pBI-CMV3-*C4BPA* in bMECs after 24 h of successful transfection, and the western blot was performed. The result showed that when bMECs were transfected with pGPU6-*C4BPA*-shRNA2, the genes that played a role in the inflammatory process, including the *IL-6* and *TLR4*, were significantly downregulated (*p < 0.05*) in the pGPU6-*C4BPA*-shRNA2 group compared to pGPU6, which is shown in Figure 6A. The expression trend of *IL-8* in the pGPU6-*C4BPA*-shRNA2 group had no significant downregulation trend compared to pGPU6, which is shown in Figure 5A. The expression of *TNF-ɑ* in the pGPU6-C4BPA-shRNA2 group was significantly enhanced (*p < 0.001*) compared to pGPU6, as shown in Figure 6A. The differing result was obtained when bMECs were transfected with pBI-CMV3-C4BPA. The genes involved in the NF-κB pathway, including inflammatory factors *IL-6*, *IL-8*, and *TLR4*, were all significantly upregulated (*p < 0.05*) in pBI-CMV3-C4BPA compared to pBI-CMV3, which is shown in Figure 5B. The expression of *TNF-ɑ* in the pBI-CMV3-*C4BPA* group was significantly downregulated (*p < 0.05*) compared to pBI-CMV3, which is shown in Figure 6B. These western blot results also validated that the expression of C4BPA had a major impact on the relative protein expression of inflammatory process related genes.

### The Relative mRNA Expression of *C4BPA* on Complement and Coagulation Cascades Genes in bMECs

**FIGURE 7:**
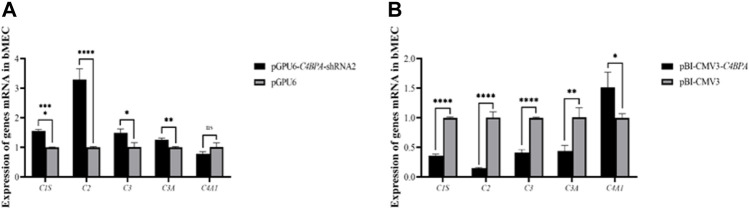
**(A)** The mRNA expression of complement and coagulation cascade pathways related genes in bMECs after transfection with pGPU6-*C4BPA*-shRNA2. **(B)** The mRNA expression of complements and coagulation cascade pathways related genes in bMECs after transfection with pBI-CMV3-*C4BPA*.

The mRNA expression of the genes related to the complement and the coagulation cascade pathways is investigated after 24 h of successful transfection with the vector pGPU6-*C4BPA*-shRNA2 and pBI-CMV3-*C4BPA* in bMECs. The results showed that when bMECs were transfected with pGPU6-*C4BPA*-shRNA2, the genes that played a role in the complement and coagulation cascade pathways, including *C1S*, *C2*, *C3*, and *C3A*, were significantly upregulated in the pGPU6-*C4BPA*-shRNA2 group, compared to pGPU6 as given in Figure 7A. The expression trend of the *C4A1* gene in the pGPU6-*C4BPA*-shRNA2 group had no significant upregulation trend compared to pGPU6, as shown in Figure 7A. The contrary result was obtained when bMECs were transfected with pBI-CMV3-*C4BPA*. The genes involved in the complement and coagulation cascade pathways, including *C1S*, *C2*, *C3*, and *C3A*, were significantly downregulated in pBI-CMV3-*C4BPA* compared to the pBI-CMV3, as given in Figure 7B. The expression trend of *C4A1* in the pBI-CMV3-*C4BPA* group had no significant downregulation trend compared to pBI-CMV3, as shown in Figure 7B. This result validates that *C4BPA* has a major effect on the complement and coagulation cascade pathways and plays a vital role in regulating these genes.

### The Effect of *C4BPA* on the Content of Triglyceride (TG) in bMECs

**FIGURE 8:**
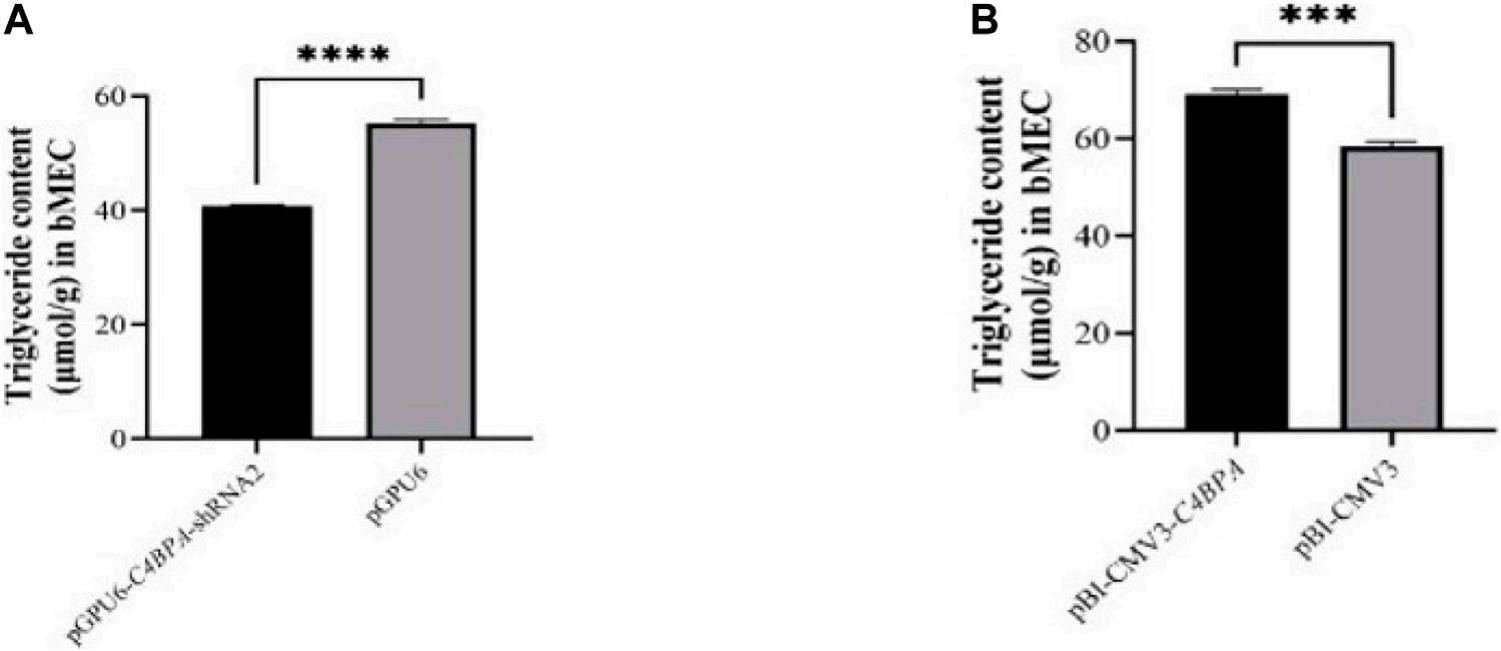
**(A)** The relative content of triglycerides in bMECs after transfection with pGPU6-*C4BPA*-shRNA2. **(B)** The relative content of triglycerides in bMECs after transfection with pBI-CMV3-*C4BPA.*

The role of *C4BPA* in triglycerides was investigated by the transfection of the vectors pGPU6-*C4BPA*-shRNA2 and pBI-CMV3-*C4BPA* in bMECs. The TG extraction was performed after 24 h of successful transfection. The result showed that when bMECs were transfected with pGPU6-*C4BPA*-shRNA2, the TG content was significantly downregulated (*p < 0.001*) in the pGPU6-*C4BPA*-shRNA2 group compared to pGPU6, which is shown in Figure 8A. The contrary result was obtained when the bMECs were transfected with pBI-CMV3-*C4BPA*. The TG content was significantly upregulated (*p < 0.001*) in pBI-CMV3-*C4BPA* compared to pBI-CMV3, as given in Figure 8B. This result validates that *C4BPA* has a significant role in the triglycerides metabolism of bMECs.

### The Effect of *C4BPA* on the Content of Total Cholesterol (CHOL) in bMECs

**FIGURE 9:**
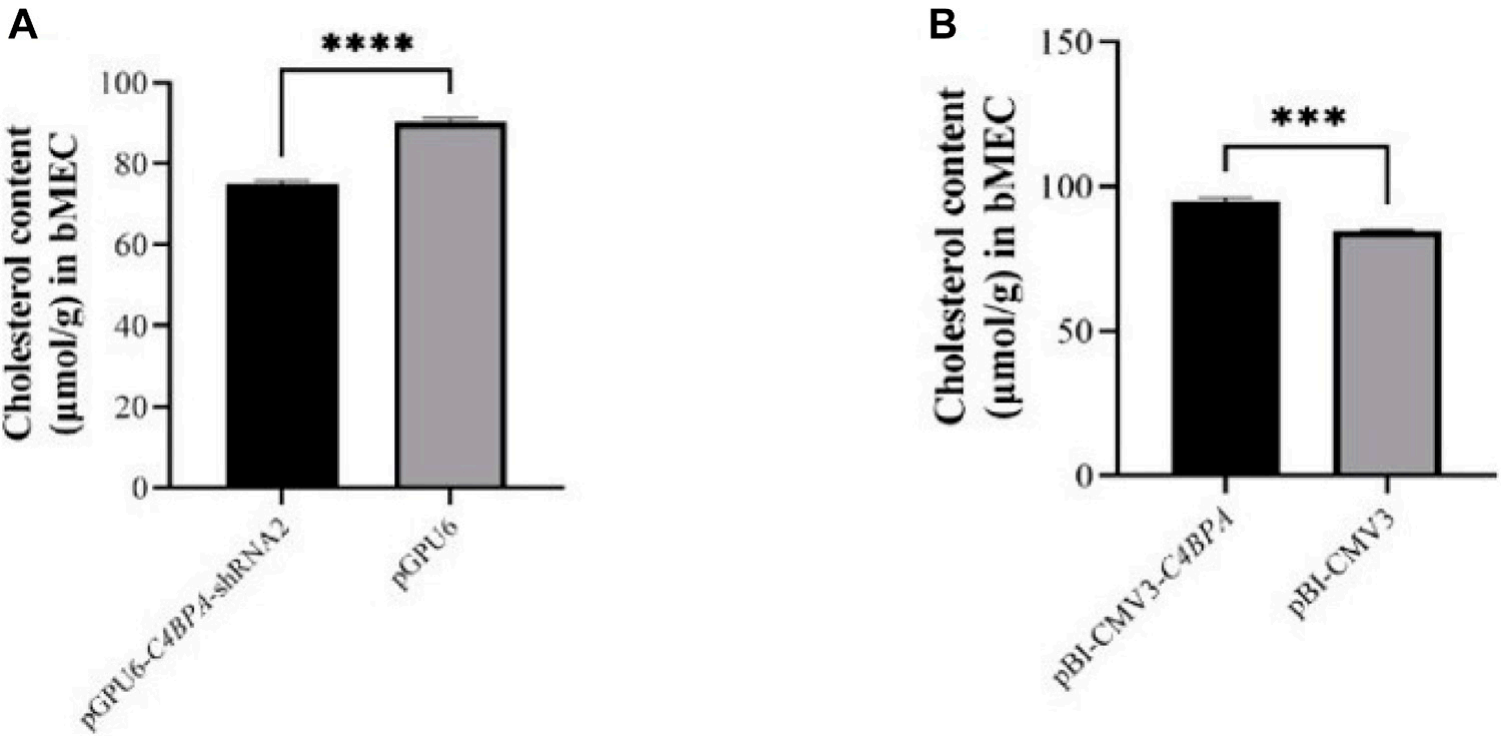
**(A)** The relative content of cholesterol in bMECs after transfection with pGPU6-*C4BPA*-shRNA2. **(B)** The relative content of cholesterol in bMECs after transfection with pBI-CMV3-*C4BPA*.

The role of *C4BPA* on total cholesterol was investigated by the transfection of the vectors pGPU6-*C4BPA*-shRNA2 and pBI-CMV3-*C4BPA* in bMECs. After 24 h of successful transfection, the CHOL extraction was performed. The result showed that when bMECs are transfected with pGPU6-*C4BPA*-shRNA2, the CHOL content was significantly downregulated (*p < 0.001*) in the pGPU6-*C4BPA*-shRNA2 group compared to pGPU6, which is shown in Figure 9A. The contrary result was obtained when bMECs were transfected with pBI-CMV3-*C4BPA*. The CHOL content is significantly upregulated (*p < 0.001*) in pBI-CMV3-*C4BPA* compared to the pBI-CMV3 shown in Figure 9B. This result validates that *C4BPA* has a significant role in total cholesterol metabolism in bMECs in addition to the TG metabolism and, therefore, it regulates fat milk metabolism.

### The Effect of *C4BPA* on the Content of Free Fatty Acids/NEFA in bMECs

**FIGURE 10:**
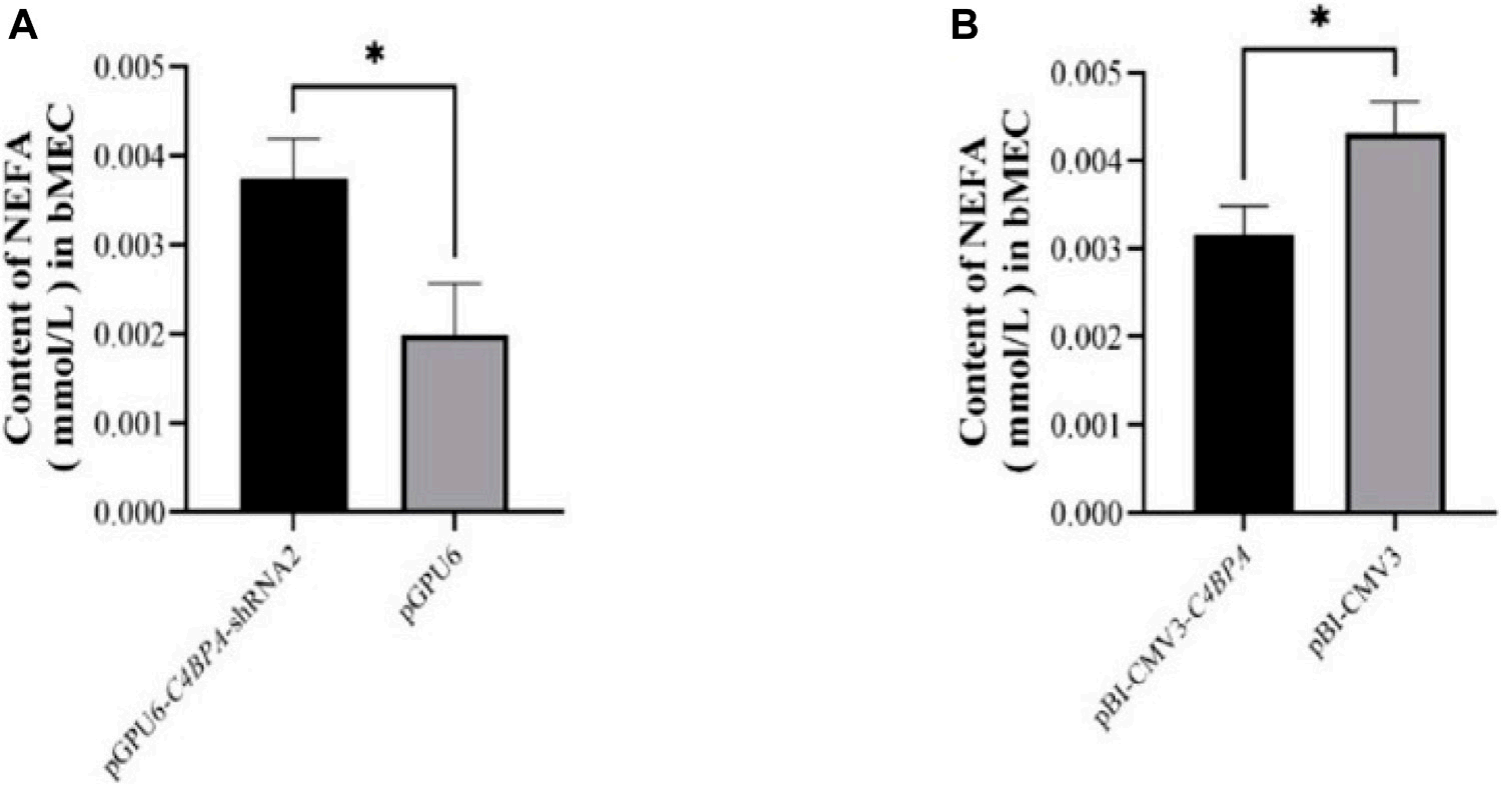
**(A)** The relative content of NEFA in bMECs after transfection with pGPU6-*C4BPA*-shRNA2. **(B)** The relative content of NEFA in bMECs after transfection with pBI-CMV3-*C4BPA*.

The role of *C4BPA* in NEFA metabolism was investigated by the transfection of the pGPU6-*C4BPA*-shRNA2 and pBI-CMV3 *C4BPA* vectors in bMECs. After 24 h of successful transfection, the NEFA was analyzed. The results showed that when bMECs were transfected with pGPU6-*C4BPA*-shRNA2, the NEFA content was significantly upregulated (*p < 0.05*) in the pGPU6-*C4BPA*-shRNA2 group compared to pGPU6, which is shown in Figure 10A. The contrary result was obtained when bMECs were transfected with pBI-CMV3-*C4BPA*. In contrast, the NEFA content was significantly downregulated (*p < 0.05*) in pBI-CMV3-*C4BPA* compared to pBI-CMV3, as shown in Figure 10B. This result validated that *C4BPA* played a significant role in NEFA metabolism in bMECs.

### The Effect of *C4BPA* on the Relative mRNA Expression of Lipid Metabolism-Related Genes in bMECs

**FIGURE 11:**
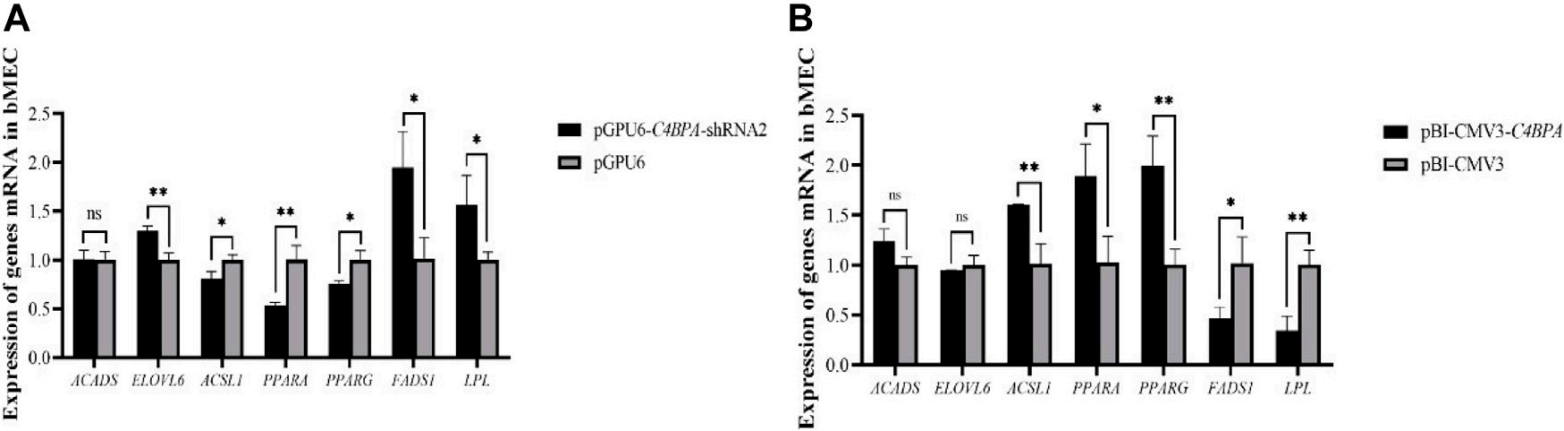
**(A)** Relative mRNA expression of lipid metabolism genes in bMECs after transfection with pGPU6-*C4BPA*-shRNA2. **(B)** Relative mRNA expression of lipid metabolism genes in bMECs after transfection with pBI-CMV3-*C4BPA*.

The mRNA expression of the lipid metabolism-related genes was investigated after 24 h of transfecting pGPU6-*C4BPA*-shRNA2 and pBI-CMV3-*C4BPA* of the vector in bMECs. The results showed that when bMECs were transfected with pGPU6-*C4BPA*-shRNA2, the genes that played a role in the lipid metabolism, including *ELOVL6*, *FADS1*, and *LPL* genes, were significantly upregulated in the pGPU6-*C4BPA*-shRNA2 group compared to pGPU6, as given in Figure 11A. The expression trend of *ACADS* in the pGPU6-*C4BPA*-shRNA2 group had no significant downregulation trend compared to pGPU6, which is shown in Figure 11A. The expression of *ACSL1*, *PPARA*, and *FADS1* in the pGPU6-*C4BPA*-shRNA2 group was significantly downregulated compared to pGPU6, which is shown in Figure 11A. The contrary result was obtained when bMECs were transfected with pBI-CMV3-*C4BPA*. The genes involved in lipid metabolism, including the *ACSL1*, *PPARA*, and *PPARG*, were significantly upregulated in pBI-CMV3-*C4BPA* compared to pBI-CMV3 (Figure 11B). The expression trend of *ACADS* and *ELOVL6* genes in the pBI-CMV3-*C4BPA* group had no significant upregulation trend compared to pBI-CMV3, which is shown in Figure 11B. Further, the expression of *FADS1* and *LPL* in the pBI-CMV3-*C4BPA* group was significantly downregulated as compared to pBI-CMV3, as shown in Figure 11B. Overall, these results authenticated that *C4BPA* has a major impact on lipid metabolism-related genes and played a vital role in regulating these genes.

### The Effect of *C4BPA* on Relative Protein Expression of Lipid Metabolism-Related Genes in bMECs

**FIGURE 12:**
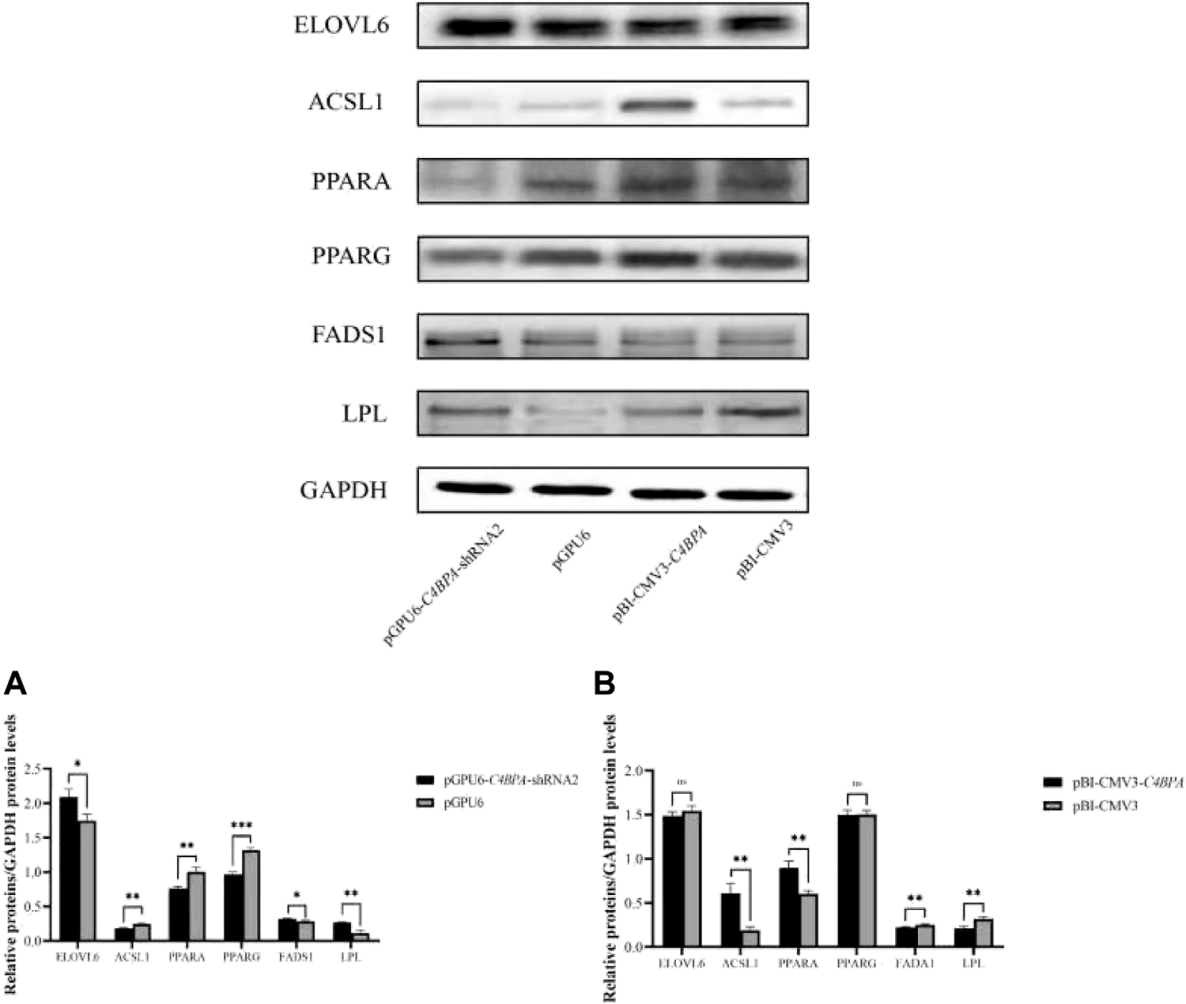
Relative protein expression of the lipid metabolism-related genes in bMECs. **(A)** Relative protein expression of lipid metabolism-related genes in bMECs after transfection with pGPU6-*C4BPA*-shRNA2. **(B)** Relative protein expression of lipid metabolism-related genes in bMECs after transfection with pBI-CMV3-*C4BPA*.

The protein expression of the lipid metabolism-related genes was investigated by the transfection of the vectors pGPU6-*C4BPA*-shRNA2 and pBI-CMV3-*C4BPA* in bMECs. After 24 h of successful transfection, the western blot was performed. The results showed that when bMECs were transfected with pGPU6-*C4BPA*-shRNA2, the genes that played a role in the lipid metabolism, including *ELOVL6, FADS1*, and *LPL* genes, were significantly upregulated in the pGPU6-*C4BPA*-shRNA2 group compared to pGPU6, which is shown in Figure 12A. The protein expression of ACSL1, PPARA, and PPARG in the pGPU6-*C4BPA*-shRNA2 group was significantly downregulated compared to pGPU6, as shown in Figure 12A. The contrary result was obtained when the bMECs were transfected with pBI-CMV3-*C4BPA*. The protein expression of genes involved in the lipid metabolism, including ACSL1 and PPARA, was significantly upregulated in pBI-CMV3-*C4BPA* compared to the pBI-CMV3, as shown in Figure 12B. In contrast, the expression trend of ELOVL6 and PPARG genes in the pBI-CMV3-*C4BPA* group had no significant upregulation trend compared to pBI-CMV3, as shown in Figure 12B. Contrarily, the FADS1 and LPL protein expression in the pBI-CMV3-*C4BPA* group was significantly downregulated compared to pBI-CMV3, which is shown in Figure 12B. Together, these results validated that the expression of *C4BPA* regulated the expression of the lipid metabolism-related genes.

## Discussion

The rapid rate of increasing human population demands an ample supply of good quality milk. One way to achieve that is to breed the dairy cows based on selecting those genes that increase milk fat and inhibit mastitis. Mastitis confreres the massive shortage of milk production and the economic loss to the dairy sector, which hinders the development of the dairy industry. Thus, cows with high-fat milk production and higher resistance to mastitis should be selected based on the molecular marker genes that play dual roles in the immunity and fat metabolism in bMECs (Waseem Ghani et al., 2019). Our previous transcriptome analysis between the high- and low-fat Chinese Holstein cattle showed that the *C4BPA* gene is differentially expressed with its higher expression in high-fat cattle. Therefore, in this study, we thoroughly explored the mechanism of the *C4BPA* gene in milk fat metabolism and mastitis in bMECs. The mammary epithelial cells can synthesize and secrete the milk components after the *in vitro* culture, so this study uses bMECs as a model (Jiang et al., 2020). The previous study reported that bMECs played a role in bacterial infection and acted as a key player in the anti-inflammatory response (He et al., 2017).

### Role of *C4BPA* Gene in Inflammatory Factors and Immunity in bMECs

*C4BPA* is a part of the extracellular complement regulator C4B binding protein (C4BP). *C4BPA* is a candidate gene for anti-mastitis and high-fat milk. The complement system is the first-line defense system that plays a key role in functioning the innate immune effectors and coordinates against the foreign invaders (Olcina et al., 2018). In contrast, the previous work demonstrated that the role of complement is mostly in tumor progression and treatment. During mastitis caused by *E. coli*, approximately within 10–12 h, the strong inflammatory response began as a consequence of chemotactic neutrophil infiltration, complement components, tumor necrosis factor, and large amount of interleukin are produced (Shuster et al., 1997). For the further progression of the inflammation toll-like receptors (TLR), NF-κB signaling plays a vital role (Khan et al., 2020). The role of the complement system in mastitis is still not explored deeply. Therefore, to validate the role of the *C4BPA* gene in the inflammatory process, the overexpression and interfering vector of the *C4BPA* gene were transfected into bMECs. This showed that when pGPU6-*C4BPA*-shRNA2 was transferred into bMECs, the expression of *TLR4* and the *NF-κB* pathway-related genes was significantly reduced. The contrary results were obtained when bMECs were treated with pBI-CMV3-*C4BPA*, in which the expression of *TLR4* and the *NF-κB* pathway-related genes was significantly enhanced. In LPS-induced mastitis, increased levels of *TNF-ɑ* and *IL-6* in the mammary gland were observed in a previous study (Ershun et al., 2014). This study reported that when the wild-type mice were treated with the lipopolysaccharides, *TLR4*, *TRAF6*, and *NF-κB* increased significantly. However, when *TLR4* knockout mice were treated with lipopolysaccharides, the mRNA and protein expression of all the genes responsible for the activation of the NF-κB pathway remained the same as the control group (Ershun et al., 2014). With the advancement of technology, it becomes evident that the protein expression of TLR4 is responsible for the secretion of the inflammatory cytokines that mediate the NF-κB signaling pathway (Tian et al., 2019). The other study through HCT116 colorectal cancer cell immunoprecipitation validated the association between *C4BPA* and *NF-κB* pathway-related genes, which showed that *C4BPA* played a significant role in the regulation of *NF-κB* dependent apoptosis (Olcina et al., 2020). These studies validated that the *C4BPA* gene played a vital role in the regulation of *NF-κB* signaling pathway-related genes. Our results also showed that when bMECs were treated with pGPU6-*C4BPA*-shRNA2, the expression of *IL-8* and *IL-12* decreased.

Moreover, the reverse trend was found in the expression of *TNF-ɑ* and *IL-10* when bMECs were transfected with pGPU6-*C4BPA*-shRNA2. However, the expression of the *IL-6* gene was not changed when bMECs were transfected with pGPU6-*C4BPA*-shRNA2. On the contrary, when bMECs were treated with pBI-CMV3-*C4BPA*, the increased expression of *IL-6*, *IL-8*, and *IL-12* was observed. In comparison, the expression of the *TNF-ɑ* was decreased in pBI-CMV3-*C4BPA*. In the early stages of infection, *IL-6* is mainly categorized as the pro-inflammatory cytokine (Tilg, Dinarello, and Mier 1997). Other researchers also reported that *IL-6* could inhibit the production of *TNF-ɑ* (Fiers 1991; Matthys et al., 1995). Similar to *IL-6*, the other inflammatory factors, *IL-10* and *IL-12*, are the critical anti-inflammatory cytokines secreted by various cells (Oliveira et al., 2017; Zhu et al., 2017). Overall, the results of this study showed that the *C4BPA* gene through *TLR4/NF-κB* played a significant role in regulating the expression of all the major genes of immunity and inflammation factors associated with mastitis in dairy cattle.

### Role of *C4BPA* Gene in Complement System in bMECs

The micro-organism is phagocytosed by the neutrophils in infected animals (Grewal, Rouse, and Babiuk 1978). This phagocytosis activity is regulated through the immunoglobulin G (IgG) and complement component 3 (C3). Despite playing an important role in phagocytosis, the complement system is also known as a non-specific humoral immune system that plays an essential role in initiating and controlling inflammation. A previous study reported that activating the alternative pathway (AP) causes more attraction and accumulation of the C3 during mastitis (Rainard and Poutrel 1995). We hypothesized that the C4BPA is a potential member of the cascade of complement and coagulation reactions. Our results indicated that when bMECs were transfected with pGPU6-*C4BPA*-shRNA2, the expression of the complement and coagulation factors *C1S*, *C2*, *C3*, and *C3A* was significantly upregulated. While the reverse result was obtained in the case of *C4A1*, in which the expression of *C4A1* was significantly downregulated in pGPU6-*C4BPA*-shRNA2 treated bMECs. In contrast, when bMECs were treated with pBI-CMV3-*C4BPA,* the expression of the *C1S*, *C2*, *C3*, and *C3A* was significantly downregulated. Contrary results were obtained in the case of the C4A1 gene that was significantly upregulated in pBI-CMV3-C4BPA transfected bMECs. Therefore, our results highlight that *C4BPA* not only involves the regulation of the inflammatory factors through *TLR4/NF-κB* but also functions as a critical player in regulating the complement system.

### Role of *C4BPA* Gene in Lipid Metabolism in bMECs

Development of the mammary glands and the onset and maintenance of lactation are incredibly complicated processes tightly regulated at multiple levels, including endocrine and molecular mechanisms (Sun et al., 2016). The lipid metabolism in bMECs is governed by many genes. Herein, we validate the role of the *C4BPA* gene in lipid metabolism. Our results indicated that when bMECs were transfected with pGPU6-*C4BPA*-shRNA2, the expression of *ACSL1* was significantly downregulated while when bMECs were treated with pBI-CMV3-*C4BPA,* the expression of *ACSL1* was significantly upregulated. A previous study reported that the overexpression of *ACSL1* was related to the upregulation of the *PPARG* gene, which enhanced the synthesis of fatty acids and lipid droplets in bovine adipocytes (Zhao et al., 2020). Our results indicated that when bMECs were transfected with pGPU6-*C4BPA*-shRNA2, the expression of *ACADS* was not changed while when bMECs were treated with pBI-CMV3-*C4BPA*, the expression of *ACADS* was significantly upregulated. *ACADS* is a member of the acyl-CoA dehydrogenase family and catalyzes the mitochondrial fatty acid *β*-oxidation pathway (Dessein et al., 2017).

Further, our results indicated that when bMECs were transfected with pGPU6-*C4BPA*-shRNA2, the expression of *FADS1* was significantly upregulated while in pBI-CMV3-*C4BPA* transfected with bMECs*,* the expression of *FADS1* was significantly downregulated. The analysis of human plasma lipid profile suggested that the fatty acid desaturase 1 (*FADS1*) has a strong association with the regulation of the polyunsaturated fatty acids (PUFA) and long-chain polyunsaturated fatty acids (LC PUFA) in human blood (Xie and Innis 2008; Glaser, Heinrich, and Koletzko 2010). Our results indicated that when bMECs were transfected with pGPU6-*C4BPA*-shRNA2, the expression of *ELOVL6* was significantly upregulated. When bMECs were treated with pBI-CMV3-*C4BPA*, the expression of *ELOVL6* was significantly downregulated. The previous study reported that the elongation of very long-chain fatty acids protein 6 (*ELOVL6*) plays a pivotal role in the energy metabolism, insulin sensitivity, and elongation of the fatty acids (Matsuzaka and Shimano 2009).

In comparison, the previous work on the bovine adipocytes suggested that the overexpression of *ELOVL6* had various effects on different fatty acid contents. The myristic acid (C14:0) and palmitic acid (C16:0) fatty acids in adipocytes decreased in the bovine adipocytes with the overexpression of *ELOVL6*. At the same time, the opposite result was obtained when the bovine adipocytes were treated with the overexpression of *ELOVL6*, and the ratio of the stearic acid (C18:0) and arachidonic acid (C20:4n6) fatty acids increased. Therefore, we speculate that *C4BPA* might regulate the fatty acid content in bMECs by regulating the gene expression of *FADS1* and *ELOVL6*. In order to authenticate the role of *ELOVL6*, our results indicated that when bMECs were transfected with pGPU6-*C4BPA*-shRNA2, the expression of *ELOVL6* was significantly upregulated. In contrast, when bMECs were treated with pBI-CMV3-*C4BPA*, the expression of *ELOVL6* was significantly downregulated. Our results also indicated that when bMECs were transfected with pGPU6-*C4BPA*-shRNA2, *LPL* was significantly upregulated while when bMECs were treated with pBI-CMV3-*C4BPA*, the *LPL* expression was significantly downregulated. Lipoprotein lipase (*LPL*) played an essential role in controlling plasma triglyceride levels. The LPL*,* through irretrievable hydrolysis of triglycerides, enhanced the removal of the triglyceride-rich lipoprotein in the plasma (Cheung et al., 2003). Another study reported that the overexpression of *ANGPTL4* in mice severely reduced the LPL-dependent plasma TG and cholesterol clearance and enhanced the cholesterol synthesis rate (Lichtenstein et al., 2007). Our results are in line with this study, and thus, we report that C4BPA, through regulating the expression of *LPL*, along with other lipid metabolism-related genes, manipulated the cholesterol content in bMECs.

The peroxisome proliferator-activated receptor (PPAR) family consisted of peroxisome proliferator-activated receptor alpha (*PPARA*), peroxisome proliferator-activated receptor beta (*PPARB*), and peroxisome proliferator-activated receptor gamma (*PPARG*). The peroxisome proliferator-activated receptor members had different distributions and functions in different tissues (Giorgino et al., 2009). The *PPARG* is a marker gene that played a significant role in early fat differentiation (Gavrilova et al., 2003) (Raza et al., 2021). Our results indicated that when bMECs were transfected with pGPU6-*C4BPA*-shRNA2, the expression of *PPARA* and *PPARG* was significantly downregulated while when bMECs were treated with pBI-CMV3-*C4BPA*, the expressions of *PPARA* and *PPARG* were significantly upregulated.

Triglycerides, cholesterol, and fatty acids are the main components of milk fat, which act as important nutrients in the organism for the synthesis of cellular components and providing essential energy (Lock and Bauman 2004). Our results indicated that the knockdown of *C4BPA* in bMECs resulted in significantly lower contents of triglycerides, and cholesterol while the content of the NEFA was up-regulated. In contrast, the overexpression of *C4BPA* in bMECs culminated with significantly higher contents of triglycerides, cholesterol and lower content of NEFA. Altogether, these results showed that the *C4BPA* gene is a major player in regulating the lipid metabolism of bMECs. This study provides the primary basis for the role of the *C4BPA* gene in lipid metabolism and immunity, but an in-depth analysis is needed for properly understanding the mechanism. This study aims to attract the researchers’ attention to focus on such type of genes that can cross-talk and affect the more important trait together. This work raises many questions, which should be considered for further extrapolation. For example, can we increase the fat milk metabolism for producing milk with high quantity of good quality fat without compromising udder health or even increasing the resistance to mastitis? Which type of association between milk fat synthesis and mastitis is present?

## Conclusion

The *C4BPA* gene involves the synthesis of triglycerides, total cholesterol, and free fatty acids acid in bMECs through regulating the expression of lipid metabolism-related genes. Additionally, the *C4BPA* gene is a candidate gene that plays a significant role in immunity by targeting *TLR4/NF-κB* and the factors involved in the complement and coagulation cascade pathways. This study reveals the dual functions of the *C4BPA* gene in bMECs in lipid metabolism and immunity. Our results provide the primary basis for understanding the role of *C4BPA* in mastitis and fat metabolism, which enables the researchers to follow the innovative direction of investigating genes associated with fat metabolism and mastitis. This research work provides crucial information for breeding cows with supreme milk quality and significant resistance to mastitis.

## Data Availability

The raw data supporting the conclusion of this article will be made available by the authors without undue reservation.
